# MYC: The Guardian of Its Own Chaos

**DOI:** 10.1002/bies.70010

**Published:** 2025-06-09

**Authors:** Abdallah Gaballa, Bastian Krenz, Leonie Uhl

**Affiliations:** ^1^ Division of Translational Cancer Research German Cancer Research Center and German Cancer Consortium Heidelberg Germany; ^2^ Chair of Translational Cancer Research and Institute of Experimental Cancer Therapy Klinikum Rechts der Isar School of Medicine Technical University of Munich Munich Germany; ^3^ Center for Translational Cancer Research (TranslaTUM) School of Medicine Technical University of Munich Munich Germany; ^4^ Department of Biochemistry and Molecular Biology Theodor Boveri Institute Biocenter Julius Maximilian University Würzburg Würzburg Germany; ^5^ Mildred Scheel Early Career Center University Hospital Würzburg Würzburg Germany

**Keywords:** Immune evasion, MYC, Replication stress, Transcription stress

## Abstract

MYC proteins are potent oncoproteins that drive tumorigenesis in a wide range of cancers, making it critical to understand their oncogenic functions and underlying mechanisms. Although MYC overexpression induces transcriptional and replication‐associated stress, recent studies have paradoxically identified MYC as a key resilience factor that protects cancer cells from these stressors. In this review, we explore the dual role of MYC in both driving and mitigating cellular stress to achieve its oncogenic function. We also examine how MYC‐induced transcriptional and replicative stress generates potentially immunogenic nucleic acid species while simultaneously helping cancer cells evade host immune recognition. We propose a model in which MYC plays a critical role in managing the stress it induces, thereby maintaining a balance that promotes tumor growth. Based on this model, we discuss potential therapeutic strategies targeting MYC‐dependent stress responses, offering new avenues for cancer treatment and highlighting the complexity of MYC‐driven oncogenesis.

## Graphical abstract

MYC is an oncogene that drives tumorigenesis through enhanced transcription and replication. Paradoxically, MYC induces transcriptional and replicative stress and acts as a resilience factor against these stressors. We discuss how MYC balances these stressors to promote tumor growth while evading immune detection and propose strategies to target MYC‐dependent stress responses.

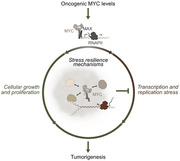

## 1 Introduction

A cell's identity is controlled by its gene expression profile, and to maintain a healthy state, gene expression must be tightly regulated. Aberrant expression of oncogenes can change the identity of a cell and promote its transformation into a cancer cell [1]. A well‐known example is MYC oncogenes, which are aberrantly expressed in many tumors due to amplification of the MYC locus or deregulated expression [2]. c‐MYC (hereafter MYC) was first identified as the cellular homolog of *v‐Myc*, the retroviral oncogene causing myelocytosis and leukemia [3, 4, 5]. Following this discovery, MYC translocations were described in Burkitt lymphoma, where MYC is juxtaposed to immunoglobulin enhancers [6].

*MYC* is a family of oncogenes, consisting of MYC, MYCN, and MYCL. These paralogs are expressed in different tissues and developmental stages, and their dysregulation can lead to different types of cancer [7]. Although aberrant expression of MYC is observed in many solid tumors as well as leukemia, MYCN is more commonly associated with neuroblastoma and other neuroendocrine tumors and MYCL is predominantly deregulated in small‐cell lung cancer. Deregulated high levels of MYC protein result not only from copy number gain or chromosomal translocation of the *MYC* gene, but also because it is downstream of other oncogenic signaling pathways. For example, KRAS activation is known to stabilize MYC, and its suppression leads to proteasome‐dependent MYC degradation [8]. Another well‐known example is observed in the WNT signaling pathway, where mutation in *APC* leads to the upregulation of MYC, thereby mediating tumorigenesis [9, 10].

MYC belongs to the basic helix‐loop‐helix leucine zipper (bHLHZ) family of transcription factors and heterodimerizes with its binding partner MAX to bind to E‐box (consensus sequence CAC(G/A)TG) DNA sequences in promoter regions, thereby activating the expression of genes involved in many cellular processes [11, 12]. MYC can regulate genes that are transcribed by the three RNA polymerases (RNAPs) [13, 14]. MYC generally activates the transcription by RNAPI and RNAPIII. However, MYC binding to promoters of genes transcribed by RNAPII can result in either activation or repression of transcription, depending on the context [15]. It is the paradigm of the field that MYC‐dependent regulation of transcription by RNAPII is the key oncogenic function of MYC, since aberrant oncogenic MYC levels can regulate coding RNA synthesis, through RNAPII, independent of growth factor availability [16, 17].

All three MYC proteins share the highly conserved bHLH‐LZ at the C‐terminus, as well as six MYC boxes (viz., 0, I, II, IIIa, IIIb, and IV). Some of these MYC boxes have well‐characterized functions and mediate binding to defined MYC‐interacting proteins. For example, MYC box I contain a phosphodegron that is important for proteasome‐dependent MYC degradation [18, 19]. MYC boxes 0 and I bind Aurora‐A, which antagonizes FBXW7 binding, thereby stabilizing MYC and MYCN [20, 21, 22]. Recently, MYC box I has been shown to be important for the ability of MYCN to directly bind RNA in vitro and in cells (discussed in Section 3.2) [23]. Other MYC boxes are important for interacting with proteins that are essential for MYC to perform its function. For example, MYC box II mediates binding to the transcription/transformation‐associated protein (TRRAP), which is a subunit of large complex proteins involved in chromatin remodeling and histone acetylation [24, 25]. It is important to note that MYC proteins are highly unstructured, with the amino terminus containing two large disordered regions [26]. Despite being highly disordered proteins, they adopt defined structures upon binding to some of their interacting partners, as shown by the crystal structure of the MYC‐MAX and MYCN‐Aurora‐A complexes [21, 27].

Although under physiological conditions, MYC proteins play important roles in transcriptional regulation, the increased transcriptional load induced by high deregulated MYC expression can cause a variety of transcriptional stresses, including DNA supercoiling and RNA‐DNA hybrids (R‐loops), which, if sustained, can lead to the formation of double‐strand breaks (DSBs) and thus genomic instability [28]. In addition, MYC has also been shown to be directly involved in promoting DNA replication, S‐phase entry, and origin firing [29, 30]. Thus, MYC overexpression causes the so‐called oncogene‐induced replication stress and induces conflicts between transcription and replication machinery [31, 32]. Paradoxically, it has also been shown that in other cellular systems (e.g., PDAC) with high levels of endogenous MYC, MYC is required to prevent transcription‐replication conflicts (TRCs) and mediate resistance to replication stress [26, 33–35]. Apart from genomic instability, high levels of replication stress can also result in the generation of immunogenic nucleic acid species that can activate innate immune signaling cascades ultimately leading to the recognition of tumor cells by the immune system [36, 37]. Although MYC overexpression contributes to this process, MYC has also been shown to have a well‐established function in masking tumor cells from recognition by the host immune system [2, 33, 38–40]. In this review, we discuss the above observations and propose a model in which MYC proteins not only exert their oncogenic function by enhancing transcription and replication, but can also protect cancer cells from the deleterious consequences of these processes.

## 2 Place MYC Under Control: MYC From Physiology to Pathology

MYC is known to control many aspects of physiological processes required by non‐transformed cells, including metabolism, differentiation, and proliferation [41]. For example, MYC plays an important role in embryogenesis as well as in the regeneration of adult tissues [42, 43]. In addition, MYC is transiently upregulated in non‐transformed cells at the G1/S transition of the cell cycle and is immediately silenced in the G0 phase of the cell cycle [44, 45]. Although MYC mediates many physiological processes in non‐malignant cells, MYC levels must be tightly regulated due to its oncogenic potential [41].

Several mechanisms have been described to regulate MYC either transcriptionally or at the RNA or protein level. MYC has a very short half‐life for its mRNA (approximately 10–20 min) and protein (approximately 20 min) [46, 47]. Several growth‐stimulating pathways, including those initiated by cell surface activation of NOTCH and EGFR, ultimately stimulate MYC expression [17, 48, 49]. On the mRNA level, MYC transcripts are rapidly turned over, but in transformed cells, they are either stabilized or undergo an increase in translation [41]. For example, MYC mRNA has been shown to be directly bound and stabilized by Argonaute 2, an RNA‐binding protein that is part of the RNA‐induced silencing complex (RISC) [50]. Post‐translational modifications of the MYC protein have been shown to regulate its stability. Phosphorylation of S62, mainly by ERK, stabilizes MYC, whereas GSK3β‐dependent phosphorylation of T58 destabilizes MYC by recruiting FBW7, which mediates its ubiquitination leading to proteasomal degradation [18, 19, 51].

If these mechanisms fail to control MYC levels, MYC also has a unique ability to limit its oncogenic potential. In non‐transformed cells, acute induction of MYC expression results in the activation of checkpoints such as p53, ARF, BIM, and PTEN, leading to cell growth arrest or death [17]. Furthermore, two common MYC mutant alleles observed in Burkitt's lymphoma have been shown to uncouple proliferation from apoptosis and are therefore more efficient in inducing B‐cell lymphogensis [52]. In addition, different thresholds of MYC have been shown to regulate its output in vivo [53]. Although low levels of MYC are able to drive the proliferation of non‐transformed cells and oncogenesis, high levels of deregulated MYC activate apoptosis and tumor surveillance pathways [53]. Thus, bypassing these intrinsic tumor suppressor functions of MYC is essential for MYC‐mediated tumorigenesis [17, 41]. In this review, we discuss the oncogenic functions of MYC in transformed cells that have bypassed these checkpoints.

## 3 Oncogenic MYC Function

Since its discovery, it has become clear that understanding the oncogenic function of MYC would be crucial for tumor therapy. Several models have been proposed to explain the mechanism by which MYC exerts its oncogenic function, which remains controversial (reviewed elsewhere [15, 54]). It has been proposed that the oncogenic function of MYC is due to its ability to bind E‐box sequences, thereby driving a specific gene expression profile required for tumorigenesis. However, although MYC binding to chromatin is nearly universal for all active genes, similar to RNAPII binding at the transcription start site (TSS), many of MYC‐bound genes do not respond to changes in MYC levels [15, 55–57], suggesting that the function of MYC likely extends beyond gene activation [15, 16, 54].

### 3.1 MYC as a Molecular Orchestrator: MYC Interactors Contribute to Its Oncogenic Function

To better understand the oncogenic function of MYC, several groups have investigated the interactome of MYC using different experimental approaches [58, 59, 60, 61]. These interactomes revealed that MYC interacts with a wide variety of factors involved in different biological functions. Based on this, the model of hand‐over regulation by MYC has been proposed [15, 16, 54]. In this model, MYC simply delivers the right cofactor at the right time and place.

For example, MYC has been shown to recruit SPT5 to the RNAPII in a CDK7‐dependent manner, thereby increasing the processivity and speed of RNAPII [61]. MYCN has been shown to recruit BRCA1 to limit the stalling of promoter‐proximal RNAPII. Mechanistically, MYCN‐dependent BRCA1 recruitment suppresses the accumulation of R‐loops (DNA‐RNA hybrids) and stabilizes mRNA decapping complexes, allowing transcription termination [62]. MYCN has also been shown to suppress R‐loop formation via Aurora‐A. MYCN activates Aurora‐A on chromatin, which phosphorylates histone H3 at serine 10, suppressing both R‐loop formation and transcriptional replication conflicts (TRCs) [34]. An alternative mechanism by which MYCN prevents TRCs is through its interaction with the RNA exosome [35]. The RNA exosome is a multisubunit complex with 3'‐5' exoribonucleolytic activity that plays an important role in transcript processing and degradation [63, 64]. MYCN recruits the RNA exosome to the RNAPII, ensuring efficient transcription elongation and preventing TRCs [35]. Taken together, MYC‐dependent co‐factor recruitment is an important aspect contributing to the oncogenic function of MYC and also opens the idea of targeting MYC‐dependent tumors by targeting its co‐factors or effector proteins (discussed in Section 5) [15, 33, 54, 65].

### 3.2 MYC Cofactors as Therapeutic Targets: Insights From PAF1c and RUVBL1/2 in PDAC

**FIGURE 1:**
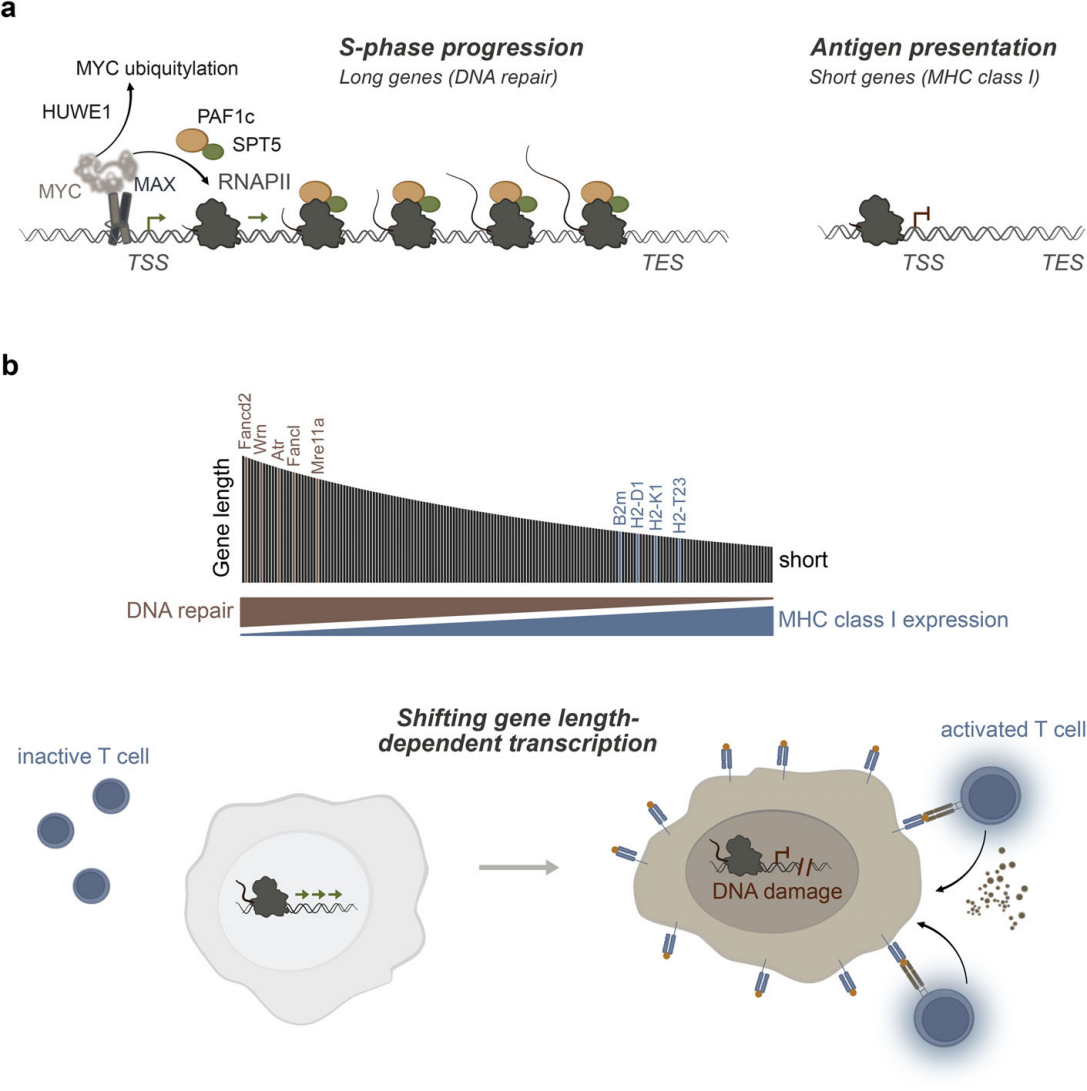
(a) MYC‐dependent PAF1c recruitment to RNAPII is essential for transcription of long DNA repair genes while limiting the expression of short antigen presentation genes. (b) Disruption of gene length‐dependent transcriptional regulation leads to increased mutational burden and genomic instability as a result of decreased expression rates of long DNA repair genes. At the same time, increased expression of short antigen presentation genes increases MHCI expression and neoantigen presentation, thereby enhancing tumor immunogenicity.

The idea that the oncogenic function of MYC might be due to its interacting proteins motivated several laboratories to test the requirement of MYC cofactors in mouse models. One of these cofactors is the polymerase‐associated factor 1 complex (PAF1c), an important transcription elongation factor for RNA polymerase II [66, 67]. PAF1c has been shown to be an important MYC interactor, and MYC has been shown to recruit PAF1c to RNAPII to promote transcription elongation and double‐strand break repair at active promoters [68, 69]. An siRNA‐mediated screen for MYC co‐factors revealed that PAF1c prevents replication stress [33]. Depletion of CTR9, a core subunit of PAF1c, in a mouse model of pancreatic ductal adenocarcinoma (PDAC) resulted in significant tumor regression, with many mice having no detectable tumor signal. This effect persisted in a subset of mice, resulting in long‐term survival benefit, and was due to T‐cell activation. Mechanistically, PAF1c sequesters SPT6 on long genes, including many DNA repair genes, to promote their full‐length transcription. This limits the presence of SPT6 on many short genes, including major histocompatibility complex (MHC) class I genes, thereby limiting their expression (Figure 1a) [33]. Suppression of MHC class I gene expression is a well‐known mechanism by which tumors evade immune cell recognition and has been known to be regulated by MYC for decades [70, 71, 72]. Consistently, PAF1c has been shown to be critical for pancreatic cancer survival in an independent study [73]. These observations suggest that genetic targeting of a MYC cofactor confers a significant survival benefit in a mouse model of PDAC and highlight the need to develop and test PAF1c inhibitors [74] for tumor therapy, particularly in tumors with deregulated MYC expression profiles. It also highlights the importance of gene‐length‐dependent transcription as a feature to be investigated in cancer.

Gene‐length‐dependent transcription depends on many factors, including SPT6 [75, 76], TFIIS [77], and CDK12 [78, 79]. Furthermore, there is evidence that this process is skewed in the context of aging [80, 81, 82], highlighting the need for precise regulation. The concept of biasing transcription based on gene length by increasing the expression of short MHC class I genes while decreasing the expression of long DNA repair genes may have the potential to impact cancer at two different levels, which may prove to be highly synergistic in eradicating tumors. First, a reduction in the expression of DNA repair genes in rapidly proliferating cancer cells that already have a high degree of genomic instability is likely to lead to an increase in mutations. Second, these mutated proteins, which are considered non‐self or neoantigens, are more likely to be presented on the cell surface and recognized by T cells due to the upregulation of short MHC class I genes [83]. Thus, this approach not only increases the pool of neoantigens, but also increases the likelihood of their presentation on the surface of cancer cells (Figure 1b).

In another study, the MYC interactome was screened for essentiality in PDAC cells and in vivo [65]. The ATPases RUVBL1 and RUVBL2 were identified as essential in both settings. RUVBL1 binding to chromatin correlated with MYC binding, and its depletion resulted in the loss of MYC binding to chromatin. In addition, RUVBL1 repressed the transcription of immune genes and activated growth genes. Genetic depletion of RUVBL1 was shown to result in significant tumor regression, preceded by immune cell infiltration. Similarly, inhibition of RUVBL1/2 with CB‐6644 resulted in tumor regression and significant survival benefit. Co‐treatment with αPD‐1 significantly prolonged the survival of PDAC‐bearing mice. Overall, RUVBL1 was required for the MYC‐dependent oncogenic gene signature, identifying this complex as a targetable vulnerability in MYC‐driven tumors.

### 3.3 MYC's Intrinsic Capabilities: Multimerization and RNA Binding

The above mechanisms demonstrate that MYC‐mediated stress resilience extends beyond that of a classical transcription factor and suggest that intrinsic capabilities of MYC may explain its oncogenic potential.

#### 3.3.1 MYC Phase‐Separation

In recent years, the biophysical principle of phase separation has become an area of intense research. In addition to well‐characterized membraneless organelles such as the nucleolus, Cajal bodies, or paraspeckles, phase separation has emerged as an important mechanism for concentrating a wide range of cellular proteins and thus plays a role in a variety of cellular processes. In transcriptional regulation, phase separation also plays an important role in concentrating RNAPII‐associated factors during all phases of transcription and has therefore been widely considered as a mechanism to enhance transcriptional output [84]. In this context, it has also been shown that several transcription factors, including MYC, undergo phase separation in vitro and that IDR‐mediated phase separation of TFs with the coactivator Mediator represents a mechanism of gene activation [26, 84, 85].

Similarly, MYCN has been shown to undergo phase separation together with MAX to boost transcription of certain genes in neuroblastoma cells [85]. These condensates include nascent RNA and components of the transcription machinery. MYCN phase separation has been shown to regulate less than 3 percent of MYCN‐regulated genes, but this set includes the activation of important oncogenes such as *SERINC2* and *ANXA8*, and has been associated with increased proliferation.

Interestingly, phase separation of MYC occurs in unperturbed cancer cells (i.e., cancer cells under normal growth conditions without external stressors) and is enhanced under various stress conditions, including proteasomal inhibition, perturbation of transcription elongation, mRNA splicing, and upon exposure to heat shock, suggesting that MYC phase separation is able to alleviate the perturbation caused by these stressors [26]. These phase‐separated multimers have been shown to play an important role in maintaining genomic stability by being recruited to stalled replication forks, allowing MYC multimers to shield stalled replication forks from RNA polymerase, thereby preventing the formation of DNA double‐strand breaks and maintaining genomic stability. MYC multimerization was shown to be facilitated by HUWE1‐mediated ubiquitylation, thereby promoting its dissociation from its promoter‐binding sites to non‐promoter regions. The ability of MYC to switch between its promoter‐bound state and its multimeric form is essential for its function in cancer cells, enabling it to overcome the transcriptional and replication stress that it encounters during rapid proliferation [26].

**FIGURE 2:**
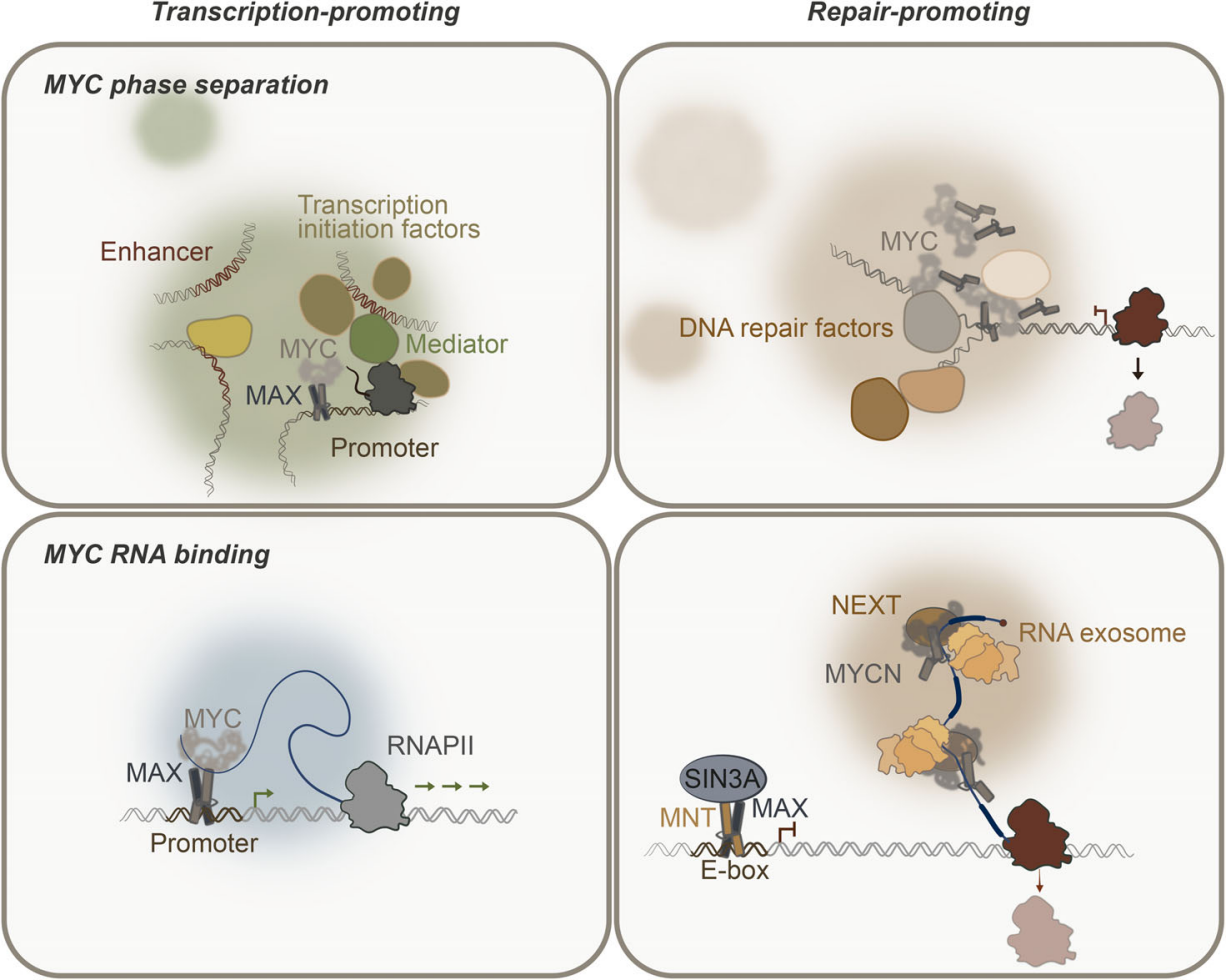
Schematic illustrating the intrinsic capabilities of MYC that contribute to its oncogenic function (adapted in part from [23]). Top left, model of how MYC phase separation enhances gene expression by concentrating transcription‐associated factors. Upper right, under transcriptional and replication stress, MYC phase separation shields transcriptional stress, thereby preventing genomic instability. Lower left, TF (e.g., MYC) RNA binding enhances transcription by promoting a dynamic interaction between RNA, DNA, and TFs on chromatin. Bottom right, direct binding of MYCN to RNA increases stress resistance during S phase by enhancing RNA processing and degradation of accumulating RNAs.

Both models integrate the fact that MYC proteins phase separate to increase the concentration of critical factors at the required site: Either to increase resilience to transcriptional and replication stress, or to activate transcription of genes important for proliferation (Figure 2).

#### 3.3.2 MYC RNA Binding

MYC proteins are well‐characterized chromatin‐associated proteins that bind to well‐characterized and defined loci throughout the genome. However, observations made by immunoprecipitation and proximity labeling experiments showed that MYC binds proteins with pleiotropic functions, many of which are associated with the transcriptional cycle, but also with DNA damage response and RNA metabolism, allowing speculation about a non‐canonical role of MYC in tumorigenesis. This paradigm shift allows to revise the view of MYC proteins, which for decades have been described only as DNA‐binding transcription factors. MYC proteins, like some other DNA‐binding transcription factors, also have the ability to bind RNA directly (Figure 2) [86].

Experiments using enhanced cross‐linking and immunoprecipitation showed that MYCN binds RNA and that depletion of EXOSC10 leads to an increase in MYCN association with aberrantly accumulating RNAs otherwise processed by the nuclear exosome [23]. This is associated with a decrease in MYCN binding to promoters, suggesting that MYCN switches from a DNA‐bound to an RNA‐bound state, presumably to limit the deleterious consequence of aberrant RNA accumulation in cells. MYCN binding to RNA was shown to be dependent on three amino acids (K51, K52, and R65) located in MYC box I [23]. Expression of MYCN in neuroblastoma cells in which these amino acids were mutated to alanine not only decreased MYCN RNA binding, but also reduced stress resilience to DNA damage during S‐phase [23]. In particular, topoisomerase I and II inhibitors showed a stronger effect on inducing DNA damage when the RNA‐binding deficient MYCN was expressed compared to wild‐type MYCN, suggesting that the RNA binding capacity of MYCN is important for stress resilience in S‐phase [23]. It remains to be tested whether the RNA binding of MYC proteins is important for tumors in an in vivo setting, and whether interfering with the RNA binding capacity of MYC proteins opens new tumor‐specific avenues to target MYC functions.

## 4 The Guardian of Its Own Chaos

MYC activation stimulates rapid cell proliferation by increasing transcription and replication. Increased transcription and replication by MYC or other oncoproteins would result in increased transcriptional and replicative stress that needs to be controlled [16, 54, 87–89]. In addition, transcriptional and replicative stress is a major source of aberrant nucleic acid species that are exported to the cytoplasm, bind to pattern recognition receptors, and activate innate immune signaling [37, 90, 91]. Thus, it is also critical that MYC provides cancer cells with the ability to evade immune cell recognition.

### 4.1 Transcription Stress

**FIGURE 3:**
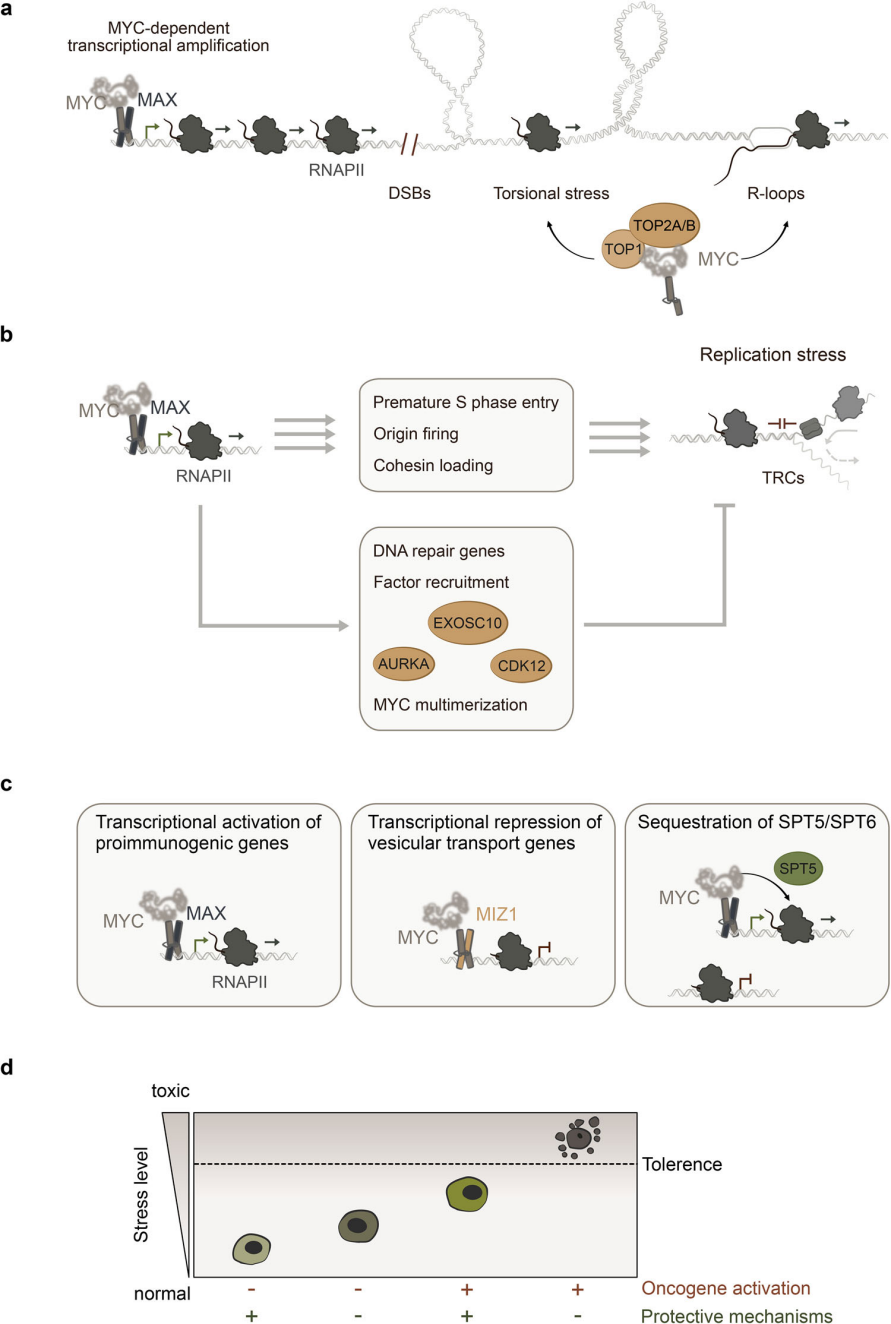
(a) MYC‐dependent transcription amplification is a source of transcriptional stress, for example, torsional stress or R‐loops, that is simultaneously counteracted by MYC‐dependent cofactor recruitment to limit genomic instability. (b) MYC overexpression leads to replication stress through premature S phase entry, allowing fining of dormant replication origins and increased cohesion loading. The resulting replication stress is counteracted by MYC‐driven resilience mechanisms such as increased transcription of DNA repair genes, cofactor recruitment, and multimerization. (c) MYC drives immune evasion by transcriptionally activating immunosuppressive genes, repressing pro‐immunogenic genes, and sequestering elongation factors away from pro‐immunogenic genes. (d) Schematic illustration of the concept of therapeutic overactivation of oncogenes. In this concept, overactivation of oncogenes stresses the cell and makes it dependent on protective mechanisms. Consequently, targeting these protective mechanisms is potentially synthetically lethal and therapeutically relevant.

Transcription by RNA polymerases is a constant source of stress that must be limited. RNAPII is responsible for the transcription of all coding genes and some non‐coding RNA. The transcription cycle consists of initiation, where RNAPII is recruited to promoters and forms the pre‐initiation complex (PIC) with other factors. This is followed by pausing of RNAPII 25–50 nucleotides downstream to assemble factors required for transcription elongation, which then occurs upon CDK9‐dependent phosphorylation of RNAPII [92], followed by transcription termination. During this process, RNAPII encounters various obstacles such as nucleosomes, R‐loops, and DNA double‐strand breaks (DSBs). In addition, during transcription, positive and negative DNA supercoiling occurs downstream and upstream of the RNA polymerase, respectively [93], which, if not relieved, can lead to DNA damage (Figure 3a).

MYC is known to be a transcription amplifier [94, 95], and this is one of the proposed models of how oncogenic MYC functions [15]. In this model, instead of activating a set of genes and repressing others, MYC amplifies the transcription of genes that are already being transcribed. In addition, MYC has been shown to stimulate RNAPII pause release, thereby increasing active transcription [96]. This high level of transcription driven by high deregulated MYC expression is likely to be stressful [97] and, if not properly relieved, will have deleterious consequences for tumor cells that require rapid proliferation.

To deal with DSBs that are likely to be formed during increased transcription, MYC recruits PAF1c to RNAPII, thereby promoting H2B ubiquitylation and allowing DSB repair [69]. In addition, increased transcriptional activity induces torsional stress. Therefore, MYC forms a complex with topoisomerases 1 and 2 to form the “topisome” and stimulates their activity to alleviate torsional stress [28]. R‐loops are considered to be transcriptional byproducts, and increased transcription leads to increased R‐loop formation [98]. Deregulated MYC expression has been shown to be associated with increased R‐loop formation and in cells with high MYC activity, topoisomerase 1 (TOP1) has been shown to regulate R‐loops and its inhibition has been shown to be synthetically lethal in MYC‐driven hypertranscription [99]. MYCN can also suppress R‐loop formation through its association with Aurora‐A and BRCA1 (discussed in 2.1). In the presence of these obstacles, RNAPII stalls during transcription elongation [100]. To cope with this, MYCN associates with the RNA exosome, allowing stalled or backtracked RNAPII to terminate transcription and allow a new transcription cycle to occur [35]. Taken together, these observations suggest that MYC‐dependent recruitment of its cofactors underlies its ability to cope with the transcriptional stress it induces (Figure 3a).

### 4.2 Replication Stress

DNA replication occurring during the S‐phase of the cell cycle ensures duplication of the DNA before cell division. Replication stress is considered a hallmark of cancer [101]. Since oncogenes, including MYC, drive rapid cell proliferation, they induce so‐called oncogene‐induced replication stress [31, 87].

MYC binds the pre‐replicative complex and directly controls DNA replication [29]. Overexpression of MYC leads to premature entry into S‐phase and increased replication origin firing, resulting in DNA damage [31]. Interestingly, using sensitive methods to map DNA replication, MYC was shown to induce the firing of novel origins of replication in intragenic positions. These dormant origins are normally suppressed by ongoing transcription in G1, but MYC activation, which induces premature entry into S‐phase, leads to origin firing within transcribed regions. As a result, conflicts between transcription and replication occur, which is associated with double‐strand breaks (DSBs). MYC‐induced replication stress has also been attributed to increased cohesin loading at specific sites in a CTCF‐dependent fashion, interfering with the progression of replication forks [32]. In addition, CDK12 has been shown to prevent MYC‐induced TRCs. Mechanistically, recruitment of CDK12 to sites of TRCs is important for repressing transcription of damaged genes, allowing for proper double‐strand break resolution [102]. Taken together, these observations suggest that MYC activation induces replication stress (Figure 3b).

Surprisingly, endogenous MYC has been shown to alleviate replication stress in a different setting [33]. MYC depletion in murine pancreatic cancer caused an increase in γ‐H2AX signal in actively replicating cells and was associated with sensitization to ATR inhibition in the induction of DNA damage. Moreover, endogenous MYC in this model was shown to limit transcription‐replication conflicts. Mechanistically, MYC‐dependent PAF1c recruitment was shown to be important for the transcription of long DNA repair genes, which are likely essential to relieve replication stress. In addition, as discussed in Sections 3.1 and 3.2, MYCN mitigates transcription‐replication conflicts through its association with Aurora‐A or the RNA exosome [34, 35], while MYC's ability to phase‐separate during transcriptional or replication stress helps protect replication forks from ongoing transcription [26].

To reconcile these seemingly contradictory observations, it is likely that MYC induces replication stress but simultaneously relieves it through faithful transcription of long DNA repair genes, co‐factor recruitment, and multimerization (Figure 3b). Interestingly, in the same model where endogenous MYC limits replication stress, overexpression of ectopic MYC induced DNA damage [33], suggesting a dual role for MYC depending on the experimental setting (discussed in Perspective).

### 4.3 MYC's Immune Evasive Potential Allows for Tumor Growth In Vivo

Increased transcription and replication can be the source of various aberrantly accumulated single‐ or double‐stranded DNA or RNA molecules that, when released from the nucleus, can activate immune signaling cascades. These so‐called damage‐associated molecular patterns (DAMPs) are therefore referred to as immunogenic nucleic acids. One example is R‐loops, the expression of which correlates with increased transcription levels and enhanced immunological responses in oncogene‐driven cancer cells [91, 98, 99, 103]. More recently, it has been shown that endonucleolytic processing of accumulated genomic R‐loops is directly linked to their export to the cytosol, where these RNA‐DNA hybrids are subsequently recognized by pattern recognition receptors (PRRs) such as cGAS and TLR3, thereby activating the observed innate immune responses [91, 98, 99]. Similarly, replication stress leading to the release of ssDNA into the cytoplasm has been linked to innate immune signaling [104]. Importantly, DNA damage resulting from both transcriptional and replication stress can also be a source of innate immune signaling through the detection of ruptured micronuclei that form after DNA mis‐segregation during cell division [90, 105]. MYC limits these problems at two different levels. First, it recruits proteins to the DNA or RNA to remove obstacles and limit stress signaling to ensure intracellular integrity and enhance proliferation, promoting a model with primarily cell‐autonomous functions. Second, there is overwhelming evidence that the contribution of MYC to tumorigenesis and its central role as an oncogene is based not only on its cell‐autonomous functions, but also on its non‐cell‐autonomous function in promoting tumor immune privilege [33, 38–40, 106–109].

To facilitate tumor immune privilege, MYC has been described to regulate multiple genes, including antigen presentation, checkpoints, chemokines, and cytokines or to modulate the metabolism of tumor cells (Figure 3c). However, the critical targets regulated by MYC vary between different experimental settings and show only a limited conservation throughout different data sets. Mechanistically, MYC was (i) described to bind to the core promoter of specific genes to promote the transcription of immune suppressive soluble signaling molecules like GAS6 or CXCL5, but also immune checkpoints like CD47 and PD‐L1 [106, 108, 109]. (ii) Other studies documented that the transcription of pro‐immunogenic genes like interferon regulatory factors and their target genes is repressed by cooperative binding of MYC and MIZ1 to the core promoter of these genes [38, 110]. (iii) The absence of MYC from the core promoter of pro‐immunogenic genes limits their transcription, while most other genes that are at least moderately bound by MYC proteins are transcribed [39, 111]. Counterselection of these genes for MYC binding limits the availability of elongation factors like SPT5 and SPT6 and favors the transcription of long, MYC‐bound genes (Figure 3c) [33]. This mechanism disadvantages genes involved in antigen presentation and favors the expression of genes that are associated with DNA repair. Indeed, MHC class I‐mediated antigen presentation by MYC proteins is well documented in multiple entities [70, 112, 113]. In a nutshell, MYC regulates the expression of crucial genes by either binding or not binding to certain genes, thereby establishing an immune suppressive signature that is conserved throughout many tumor entities (Figure 3c) [40, 114].

Beyond the regulation of specific pro‐immunogenic genes, MYC restricts the activation of TBK1, a kinase that integrates the signaling of multiple pattern recognition receptors. The deletion of TBK1 in tumor cells reverts the survival benefit after depletion of MYC in a model of pancreatic cancer [39]. To limit the signaling of innate immune receptors to TBK1, MYC suppresses the formation, metabolism, and transport of immunogenic dsRNA [39, 115]. The cGas/STING axis is sensing DNA fragments or R‐loops in the cytosol, both are a common consequence of defects in the DNA damage repair mechanisms. MYC limits the expression of STING proteins thereby rendering tumors blind to immune activation upon DNA damage [116, 117]. In line with that, MYC represses the transcription of pro‐immunogenic genes downstream of cGas/STING signaling [110]. Classically cold MYCN‐driven neuroblastoma predominantly present without a functional cGas/STING axis, underlining, that taming this pathway is crucial during MYCN‐driven tumorigenesis [118].

MYC allows tumor cells to evade the immune system on two different levels: First, MYC intracellularly limits the accumulation of stress and damage‐associated molecular patterns and controls the challenges that come along with tumorigenic proliferation. Secondly, oncogenic MYC levels favor via diverse mechanisms the repression of pro‐immunogenic signaling and promote immune evasion in multiple entities. In transformed cells, promoting immune evasion is a prerequisite for MYC to mediate its strong potential as an oncogene and to allow the outgrowth of intrinsically challenged and stressed tumors in patients.

## 5 Targeting MYC: Challenges and Opportunities

MYC proteins orchestrate and regulate manifold processes ‐ from the nucleus to the plasma membrane of eukaryotic cells and its omnipresence in human tumors make it a tempting target for tumor therapy. Though, MYC proteins are widely considered to be challenging to the drug for three major reasons: first, MYC proteins are not exclusively expressed in tumor cells, but are also expressed in hematopoietic cells and proliferating or regenerating healthy tissue. Targeting all functions of MYC would cause severe side effects and does not promise benefit for patients. Second, MYC proteins are transcription factors lacking enzymatic activity and its disordered structure makes it difficult to design small molecules. Manifold attempts to target MYC or its function, as well as Achilles heels of MYC‐deregulated tumors in cells have been tested in cell systems, in vivo mouse models, or are even now in clinical trials. MYCi that interfere with the MYC/MAX complex reduces tumor growth and synergizes with immunotherapies and has well tolerable side effects [119]. Along that line, the application of the 91 amino acid mini‐protein OMO‐13, a derivate of the dominant negative MYC allele “OMOMYC”, has been successfully tested in clinical trials, inducing stable disease in some but not all patients [120].

However, the field has identified several synthetic lethalities in MYC or MYCN‐deregulated tumors. Some of those, like the combination of ATR (AZD6738) and AURAKA (MLN8237) inhibition or targeting RUVBL1/2 (CB‐6644) are validated in vivo with compounds [34, 65]. Others, like the targeting of the exosome in MYCN‐driven neuroblastoma or CTR9 in PDAC are still to be validated using compounds in vivo, but are still promising targets that promise less toxicity and better drugability than MYC proteins itself [33, 35]. Similarly, targeting the interface between MYC and MIZ1 to disrupt the complex that contributes to the downregulation of certain pro‐immunogenic genes is likely to increase immune visibility of tumor cells in vivo [38, 39, 121]. However, inhibitors, that directly target the interaction of MIZ1 and MYC are yet to discover. The interaction between MYC and MIZ1 is only present at supraphysiological levels of MYC, allowing for targeting a purely oncogenic function of MYC.

Very recently, MYC proteins function and biochemistry extends beyond binding to chromatin toward multimerization and binding to RNA in the cell. This indeed allows for targeting highly specific multimeric structures of MYC that are facilitating stress resilience [23, 26]. Degrader molecules or drugs that interfere with these high molecular weight complexes are yet to be identified.

### 5.1 Perspective

The aforementioned observations show that MYC rewires cell mechanisms of stress resilience to meet its need for uncontrolled rapid proliferation. An obvious strategy in targeting MYC's oncogenic function is to target its protective functions while sparing its roles in driving cellular growth and proliferation. By doing so, rapidly proliferating cancer cells will become susceptible to disruption of their stress resilience mechanisms. This strategy may also be less toxic, as it would minimally affect healthy highly proliferative cells.

For example, TOP1 was shown to suppress R‐loop accumulation in MYC‐transformed cell lines, and its inhibition or genetic depletion reduced the fitness of MYC‐transformed tumors [99]. Another example is the inhibition of Aurora‐A, a co‐factor of MYCN that suppresses R‐loop formation, which induces transcription‐replication conflicts and activates ATR kinase to limit DSBs. Co‐treatment with an ATR inhibitor results in tumor eradication of MYCN‐driven neuroblastoma and shows no evidence of toxicity to adjacent or highly proliferative tissues, most likely affecting only tumor‐susceptible tissues [34]. Similarly, depletion of PAF1c, a cofactor of MYC that ensures the transcription of DNA repair genes and suppresses MHC class I genes, results in complete tumor eradication in a subset of PDAC‐bearing mice [33]. It is highly likely, but remains to be tested, whether MYC's intrinsic ability to phase separate and/or bind RNA contributes to its oncogenic potential in a relevant tumor model. Thus, targeting MYC co‐factors or its intrinsic properties that limit MYC‐induced stress may be an attractive way to therapeutically target MYC‐deregulated tumors.

### 5.2 Step on the Gas and Take Off the Helmet: Therapeutic Overactivation of Oncogenes

Recently, the concept of “therapeutic overactivation of oncogenes” has emerged, which aims to activate oncogenes beyond the stress threshold that cancer cells can manage (Figure 3d) [122, 123]. For example, inhibition of protein phosphatase 2A (PP2A) upregulates several oncogenes, including MYC, which then induce stress responses. As a result, tumor cells become susceptible to inhibition of the mitotic gatekeeper WEE1, leading to collapsed replication, premature mitosis, and ultimately cell death.

Building on this concept, overexpression of MYC in PDAC cells (which already have high levels of MYC) significantly increases the percentage of cells in S‐phase, which is associated with increased DNA damage [33]. Under these conditions, MYC‐overexpressing cells become more dependent on protective mechanisms, as PAF1c depletion significantly reduces their ability to actively replicate DNA, accompanied by a significant increase in DNA damage and apoptosis [33]. Importantly, PAF1c depletion in control cells without MYC overexpression had minimal effects on cell cycle progression and DNA damage induction. Taken together, these findings suggest that cancer cells with high levels of oncogene expression are more dependent on their protective mechanisms to cope with the stress they induce. Consequently, targeting these mechanisms may be a relevant therapeutic approach (Figure 3d).

## Conflicts of Interest

The authors declare no conflicts of interest.

## Data Availability

Data sharing is not applicable to this article as no datasets were generated or analyzed during the current study.
